# A systematic review on the application of precision livestock farming technologies to detect lying, rest and sleep behavior in dairy calves

**DOI:** 10.3389/fvets.2024.1477731

**Published:** 2024-12-23

**Authors:** Gaia Pesenti Rossi, Emanuela Dalla Costa, Sara Barbieri, Michela Minero, Elisabetta Canali

**Affiliations:** Department of Veterinary Medicine and Animal Sciences, University of Milan, Lodi, Italy

**Keywords:** dairy calves, lying, rest, sleep, PLF, sensor

## Abstract

Welfare studies are increasingly involving the application of Precision Livestock Farming (PLF) sensors, rather than the use of animal-based indicators directly assessed. PLF technology has the advantage to constantly monitor behavior over a long period of time, thus enabling the assessor to identify changes in animal time budgets in real-time. In calves, lying behavior is essential: new-borns have been reported to spend 70–80% of their daily time lying. Growing up, calves progressively reduce the time spent lying; at 3 months, lying behavior occupies around the 50% of their day. Several studies emphasize how lying behavior can be considered as a potential indicator of positive welfare in ruminants, including calves. The aim of this study was to critically revise scientific literature regarding the application of precision livestock farming technologies to measure lying, rest and sleep behaviors in dairy calves. A systematic literature search based on the Preferred Reporting Items for Systematic Reviews and Meta-Analyses (PRISMA) methodology was conducted through Scopus and Web of Science databases to retrieve full peer-reviewed papers written in English on different PLF technologies applied to measure lying behavior in dairy calves. Literature search retrieved 731 records. After duplicate removal and the application of inclusion criteria, a total of 16 papers were considered eligible for the evaluation. Different PLF technologies and approaches were reported to be used: triaxial accelerometers, machine learning with accelerometer data, computer vision with video cameras, wearable cameras and real-time locating system. Most of the papers (10 out of 16) reported the use of accelerometers, placed on different parts of body of the animal (hind leg, neck, head, ear). Considering the importance that lying behavior has for maintaining homeostasis and development of calves, the possibility to monitor it constantly and reliably with PLF technology would certainly provide a better understanding of calves’ behavior and positive welfare. However, our findings underline PLF technologies still show some practical limitations. Therefore, we must ensure that the sensors are valid and reliable before applying them in practice to detect changes that can be linked with welfare status of calves.

## 1 Introduction

The welfare of farmed animals is increasingly an issue of interest to citizens and consumers (1), therefore the evaluation of the welfare of farmed animals is an important and practical research field. In recent years, the approach to welfare assessment has been moving from the use of animal-based indicators related to poor welfare, highlighting negative emotions (2), to the search for indicators that can highlight the positive states of animals (3–5). Among them, studies have emphasized the potentiality of indicators in ruminants such as lying time, frequency and duration of bouts and synchronicity in lying in dairy cattle and in calves (6, 7). Lying behavior, in particular the increased time budget over 24 h, the possibility of performing adequate postures and the ease of getting up and lie down, have been recently described as environment-induced component of positive welfare, given the importance of comfort and appropriate rest in ruminants’ life (6). Indeed, lying time was suggested as positive indicator for assessing bedding quality and thermal comfort, as it has been observed that lying time increases in dairy calves provided with a more insulating substrate [e.g., sawdust rather than river stones for calves (8)]. Moreover, the role of rest and sleep in dairy calves’ development and overall welfare is crucial, as reported also in humans and other animals, particularly growing ones (9, 10). In fact, sleep contributes to the growth and development of young animals by regulating the secretion of several hormones such as GH and glucocorticoids (11) and is essential for brain development (10, 12).

Time spent lying is very important for calves: newborn calves have been reported to spend lying 16–18 h per day, mainly while lying on the sternum, occupying about 70 to 80% of the day (13, 14). The time spent lying is reduced as the calves grow, and this behavior occupies approximately 50% of the day during the first 3 months of age (14), while feeding behavior increases (15–17). However, the rate of decrease in lying behavior with increasing age could be affected by the housing and feeding systems (13, 18, 19). It has also been reported that the duration of lying behavior may be an adequate indicator of the health and welfare of calves (20, 21). Increased lying time, in particular, is thought to help conserve energy for mounting an immune response (22): in the work of Borderas et al. (23) for example, dairy calves injected with bacterial endotoxin (LPS) spent more time lying inactively. Similarly, Cantor and Costa (24) reported that calves affected by bovine respiratory disease (BRD) increased their lying times during the 5 days before BRD diagnosis and on the day of diagnosis compared with healthy calves. Moreover, lying behavior has also been studied in calves as an indicator of adaptation to new housing systems and overall comfort (25, 26).

When calves are resting and sleeping, they usually lay down and use several postures including one in which they rest with the head on the legs and another in which the legs are fully stretched out (27, 28). Moreover, Hanninen et al. (28), observed that sleeping behaviors are relatively good measures for the total sleeping rhythm and the overall time spent of NREM and REM sleep.

Lying measurements, like the quantification of time spent lying down within a period of time (24 h) and the frequency and duration of lying bouts (i.e., the transition between lying and standing), can be automatically acquired through precision livestock farming (PLF) technologies, which are now commonly used in dairy cattle (29, 30), although not as widely used for calves. Moreover, while data on lying time in calves is available, there is scarce information distinguishing between rest and sleep, as also reported for adult bovines.

This review aims to evaluate the approaches reported in the literature regarding the application of precision livestock farming technologies to measure lying, rest and sleep behaviors in dairy calves, in order to monitor their baseline and detect potential alterations.

## 2 Materials and methods

A systematic literature search based on the Preferred Reporting Items for Systematic Reviews and Meta-Analyses (PRISMA) methodology was conducted.

### 2.1 Inclusion and exclusion criteria

Peer-reviewed scientific articles describing the use of sensors for detecting lying, rest and sleep behavior in healthy calves, defined as bovines with less than 6 months of age, were eligible for inclusion. Literature search was conducted through Scopus and Web of Science on 13 November 2023, to include articles written in English, with full text available and based upon original data.

Since this critical evaluation focused on the use of sensors to detect lying, rest or sleep behavior in calves, all studies evaluating other animal species or adult bovines, as also the alterations of these behaviors depending on management practices or the presence of disease in calves were excluded.

### 2.2 Search strategy

The search was conducted applying the following search terms: (calf OR calves OR dairy cal* OR veal cal* OR young cattle) and (sensor OR accelerometer OR activity sensor OR artificial intelligence OR computer vision OR non-invasive technology OR logger OR machine learning) and (rest* OR sleep* OR lying). The selection of these search terms was based on initial screening of relevant articles to gain general background information and expert opinion. The terms were searched within article title, abstract and keywords.

### 2.3 Screening and data extraction

All papers obtained from the database searches were exported into a Microsoft Excel file, and a first cleaning of the dataset was conducted to remove papers that did not report the authors’ names or abstract and to maintain only peer-reviewed papers. Before screening, all duplicates were removed. The screening process was carried out by the first author (G.P.R.). Firstly, any record with a title that clearly did not fit the eligibility criteria was excluded. The remaining papers were screened based on the abstract, and subsequently on the full text. The information regarding animal age and weight, type of sensor and site of application (when wearable), type of behavior recorded, sampling interval and other measures taken for the study, were tracked and tabulated.

## 3 Results and discussion

**Figure 1:**
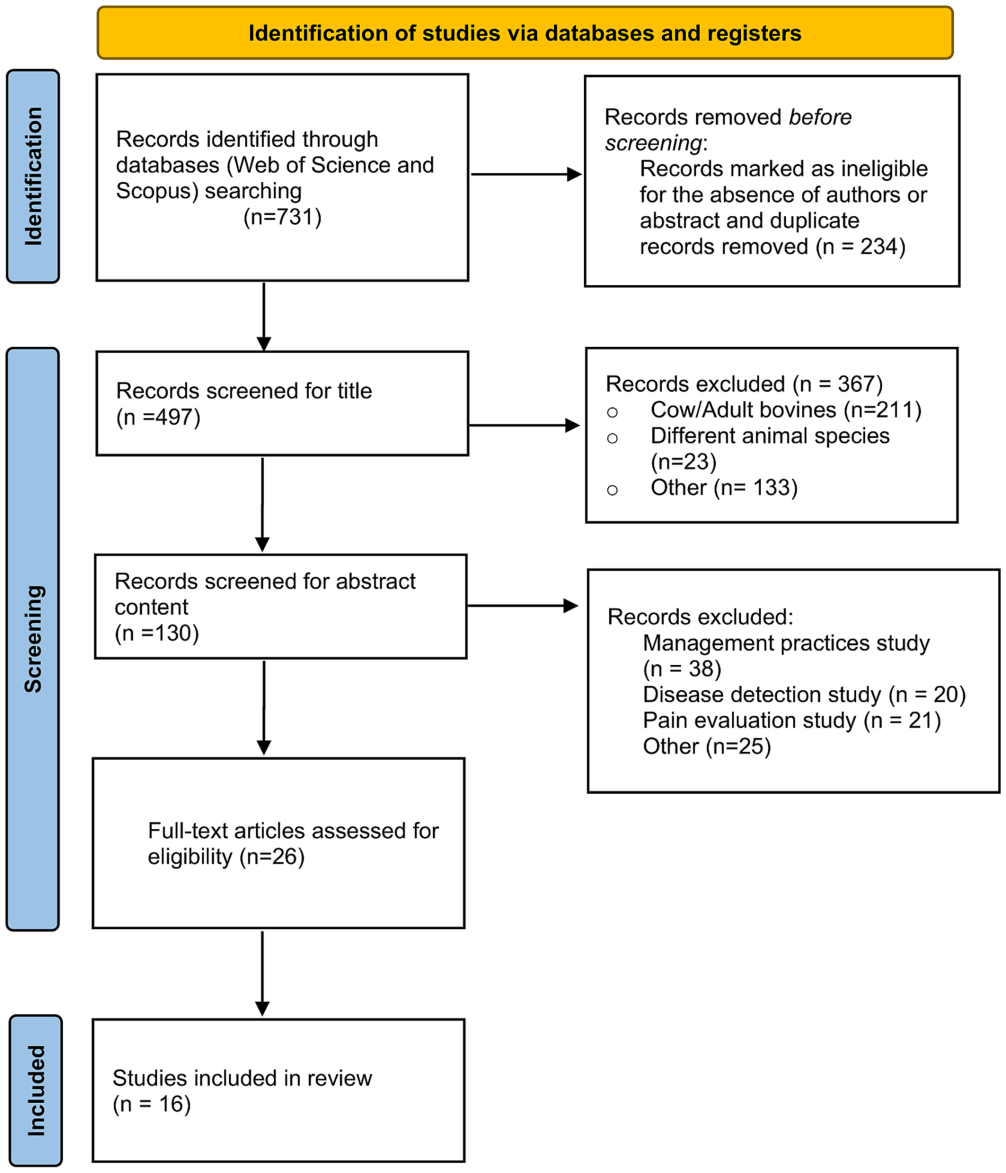
A modified PRISMA flow diagram provides information on the number of excluded papers and the reason for exclusion.

Literature search returned 731 records. After duplicate removal and the exclusion of papers without authors or abstract, a total of 234 records were eliminated. Subsequently, 481 papers were excluded, according to the application of inclusion criteria throughout the review process. A modified PRISMA flow diagram provides information on the number of excluded papers and the reason for the exclusion (Figure 1). A total of 16 papers have been included in the present review and will be discussed according to the type of sensor or technology used in the study. Moreover, the description of the behavior under study will be further detailed and discussed.

Table 1 summarizes the features of the included study, including the research topic, the animals included in the study (number, breed and age) and their housing, the timing of the study, the validation with visual observation or video recordings and the site of the study.

**Table 1:** Features of the included study, including the research topic, the animals included in the study (number, breed and age) and their housing, the timing of the study, the validation with visual observation or video recordings and the site of the study.

Paper	Research Topic	Animals included (n°; breed, age)	Housing	Timing of the study	Validation	Research site
(31)	Tri-axial accelerometer	9 Holstein calves (3 male and 6 female), 2 months of age	Group-housing, 2.49×4.14 m pens, 4 calves/pen	12 h period: from 09.00 to 21.00	Videorecording	Not specified
(20)	Tri-axial accelerometer	Experiment 1: 8 dairy calves (4 male, 4 female), 21.5 ± 14.5 days old. Experiment 2: 19 female dairy calves, 29.4 ± 4.6 days old.	Experiment 1: individual housing (4 calves, 2.5 × 1.4 m); group-housing (4 calves, 4 × 2.5 m, 5 calves/pen) Experiment 2: group-housing (10 × 4 m; 25 calves/pen)	Experiment 1: 37 × 2-h observation periods (27 × 2 h of individual-housed calves; 10 × 2 h of group-housed calves). Each calf was observed for 4 ± 2 periods of 2 h. Fitted with 3 data loggers for 4 ± 1 d. Experiment 2: 24 h-recording period	Experiment 1: Visual observation Experiment 2: Videorecording	Experiment 1: Experimental station Experiment 2: Commercial dairy farm
(21)	Tri-axial accelerometer	5 unweaned female calves (2 Jersey and 3 Holstein), 44.6 ± 3.2 days old	Group-housing	Sensors worn for 10 days. 7-h period during daylight hours for video analysis	Videorecording	Experimental station
(33)	Tri-axial accelerometer	13 Holstein calves (7 male, 6 female), 55 ± 5 days old	Group-housing, 6 × 6 m pen, 20 calves/pen	Sensors worn for 4 days (96 h)	Videorecording	Not specified
(34)	Tri-axial accelerometer	15 female Holstein-Friesian calves, 39 ± 8 days old	Group-housing, 7.90 × 3.90 m, 15 calves/pen	Videorecording between 07.00 and 11.00, thus the behaviour of each calf was video classified for 4 h, resulting in 60 h classification data. After nonidentifiable behavior, resulted 38 h 7 min of usable observation time in total	Visual observation, videorecording	Commercial dairy farm
(35)	Tri-axial accelerometer	5 pre-weaned dairy calves, 53 ± 20 days old	Individual housing, 122 × 46 cm	Loggers’ data collection from 07.00 to 08.36, in 1 day	Visual observation	Experimental station
(36)	Tri-axial accelerometer	8 female Holstein calves, 30 days old	Individual housing	Data logging for 6 consecutive days	No	Commercial dairy farm
(37)	Tri-axial accelerometer	10 calves, 31.7 ± 5.4 days old	Group-housing, 5.3 × 3.6 m	Recording session of 24 h	Videorecording	Commercial dairy farm
(38)	Tri-axial accelerometer	15 female Holstein calves, data collected since they were 0.5 months old	7 types of housing at different ages	Data collected at 0.5, 1, 2, 4, 8, 12, and 18 months, and at 23 months or 1 month before the first calving	Visual observation	Experimental station
(32)	Tri-axial accelerometer	16 male Holstein calves, 4 to 6 weeks of age	Individual housing, 1.2 × 2.4 m	Instantaneous recording was applied at 1-min intervals (5 to 10 s/calf each min) for 12 h/d on 4 different days	Visual observation	Experimental station
(39)	Machine learning with accelerometer data	15 calves	Group housing	4 h data collection per calf	Videorecording	Not specified
(40)	Machine learning with accelerometer data	13 Holstein calves, 5–7 weeks old	Group-housing, 6 × 12 m, 20 calves/pen	10 days data collection	Videorecording	Experimental station
(41)	Computer vision with video camera	1 Holstein calf, 2 months old	Individual housing, 4 × 2 × 1.5 m	Image/video data were collected from 07:00 to 18:00 h each day in July 2013	Videorecording	Commercial dairy farm
(42)	Computer vision with video camera	Number of animals included is not specified, but each image consists of at least two calves clearly visible. Holstein-Friesian Cross breed	Individual housing	The images were being collected for 2 months continuously with a 5-s interval in between each frame from the camera	Videorecording	Experimental station
(43)	Wearable cameras	4 female Holstein calves, approximately 2 months old	Group-housing, 6.96 × 8.41 m, 4–7 calves/pen	Calf behavior was recorded every 30 s using a wearable camera from 10:00 to 15:30 and observed directly from 11:00 to 12:00 and 14:00 to 15:00	Visual observation, Videorecording	Experimental station
(44)	Real-time locating system	5 female Holstein calves, 31 ± 6.2 days old	Group-housing, 5×11 m	Recording coordinates with a frequency of 5 Hz for 30 days	Videorecording	Experimental station

### 3.1 Tri-axial accelerometers

Among the 16 papers included, 10 articles dealt with the use of tri-axial accelerometers. This type of sensor measures the acceleration forces towards the three axes (x, y, z) which occurs during animal movement at determined sampling intervals, and then statistical analysis or algorithm were applied to translate the measurements into posture and behavior output files.

Seven papers (20, 21, 31–35) quantified the time spent lying (both in percentage of the day and as the duration in minutes), referring to the sole posture of the animal, and quantifying the so-called “bout,” defined as the transition between lying and standing, also estimated in terms of mean duration and frequency in the considered timespan. We can notice that this aspect defines only the posture of animal and is generally linked to a state of inactivity or rest. It is therefore indirectly related to resting behavior and sleep. For this kind of evaluation, accelerometers were placed on the right hind leg as a pedometer (20, 21, 31, 33, 35), or on the ear as an ear tag (32, 34).

Temporal distribution of resting activity was deduced by the measurement of total locomotor behavior recorded by activity data logger in the work of Giannetto et al. (36).

On the other hand, two papers focused on the specific evaluation of sleep (37, 38) and its stages (37). As previously reported by Hänninen et al. (28), in fact, sleeping behavior and its stages can be defined as follows: NREM sleep when the calf is resting head up, being still, and REM sleep when the calf is resting neck relaxed, with the head against the floor or flank. Considering the implication of head and neck in sleep evaluation, accelerometers were placed, respectively, on the middle occipital using a halter and on a collar in the works of Hokkanen et al. (37) and Fukasawa (38).

Within the reviewed studies, sensors could be differentiated based on their applications: specifically, sensors can be categorized into those with research features (R), such as customizable sampling frequency and the ability to download and analyze data, and those designed for on-farm use with real-time software applications, referred to as ‘commercially available sensors’ (C).

In particular, considering the different commercial accelerometers found in literature search, it must be noted that none of them was purposely designed for calves: it was indeed used a sensor which had been previously validated on cows. This implies that the features of the sensor are relevant for their practical use on calves: when considering pedometer and ear tag, in fact, sensors must be light and little enough, not to cause disturb to animals, both for their welfare, but also for the quality of data acquired from technology.

The distinction based on the application of accelerometers also relates to real-time availability of data and the necessity of subsequent data elaboration (R) or automatic algorithm application (C).

Hobo Pendant G logger (R) was the most reported sensor in literature (4 papers out of 10): it was positioned as pedometer and validated for recording lying behavior of unweaned calves considering different anatomical site (i.e., forelimbs and hind limbs (20), but also considering a different environmental condition [i.e., validation in tropical weather (35)]. It was also positioned middle occipital on a halter and validated for measuring sleep-like postures as indicator of sleep behaviors (38). Moreover, it was also considered in the work of Swartz et al. (21), its data for measuring lying behavior were also used to compare and validate the sensor object of the paper (i.e., Afitag II). In all the aforementioned cases sampling frequency was decided by the authors and after the experimental period, data were downloaded to a computer using HOBOware Pro Software and subsequently elaborated with statistical programs (i.e., SPSS, R, SAS, and Excel).

On the contrary, data from C sensors found through literature search (namely Afitag II, IceQube, Icetag, Actiwatch Mini, SmartBow and SensOor) were automatically elaborated through private algorithm (unpublished), resulting in a potentially prompt instrument for on-farm application, while fewer information are available about how behavioral quantification was carried out. However, all the above-mentioned sensors were validated with visual observation or video recordings. Considering that raw data are not available and summarized at intervals of 15 min (as for Afitag II and IceQube), validation was made based on the sum of data, aligned with the timing of video recordings or visual observations.

### 3.2 Machine learning with accelerometer data

Literature search also resulted in 2 papers (39, 40) focused on the approach and the algorithm used to classify and quantify multiple behaviors using accelerometer data, rather than focusing on the sole sensor and on one type of behavior. This implies a rising interest in the integration of different types of behaviors, using algorithms with the attempt of describing the complexity of animal behavior (also considering the implication of its alteration). Lying behavior was considered and most represented, and one of the two papers (40) also evaluated aspects of active and non-active lying with high levels of accuracy (90.38% both) although active lying had the worst performance in terms of sensitivity (64%) and precision (69%), probably because it was confused with similar behaviors as non-active lying or ruminating. Providing more detailed information about the level of activity performed while lying and what the calf is actually doing, is a largely unexplored aspect considering the results of the present review, and a reason might be linked to the necessity of finding sufficiently sensitive sensors.

Sturm et al. (39) applied a chaos theoretic approach to animal activity recognition: animal behavior was considered as a nonlinear dynamical system and time series derived from ear-tags with tri-axial accelerometers [Smartbow, previously validated by the same authors in the work of Roland et al. (34)] were used to associate two main mutually exclusive behaviors, namely, lying and standing/walking with six possible activities: feeding, drinking water, drinking milk, playing, rumination, and neutral behavior.

Carslake et al. (40) developed an algorithm to identify postures (namely lying and standing) and several behaviors (i.e., active lying, non-active lying, locomotor play, self-grooming, ruminating, non-nutritive and nutritive suckling) in 13 pre-weaned dairy calves with collar-mounted accelerometers (Sparkfun 9, research-based). In particular, a quantification algorithm for predicting behavior distribution was developed and validated. This approach, referred by authors as mostly ignored in precision livestock, has the advantage of showing high accuracy with relatively low overestimation in unseen real-world data despite very low behavior prevalence. The identification of rare behaviors might be of strong interest, considering its use for disease and welfare prediction and quantification.

Table 2 presents detailed information about the accelerometers used in tri-axial accelerometer studies and machine learning studies involving accelerometer data. It includes specifics on the sensor’s placement, its application, the type of behavior or posture recorded and the related behavioral measures, as well as data sampling frequency and elaboration.

**Table 2:** Information about the accelerometers used in tri-axial accelerometer studies and machine learning studies involving accelerometer data, regarding specifics on the sensor’s placement, its application, the type of behavior or posture recorded and the related behavioral measures, as well as data sampling frequency and elaboration.

	Tri-axial accelerometer	Position of the sensor	Research (R) /Commercial (C)	Type of behaviour/posture	Behavioural measures	Data sampling frequency	Data Elaboration	Paper
Tri-axial accelerometers	IceTag	Right hind leg, above the fetlock	C	Lying	Lying (% lying) Standing (% standing) Moving (% active)	8 Hz (data obtained on a 1-s basis)	Data downloaded from IceTag device to a computer and processed using IceTagAnalyzer software	(31)
	Hobo Pendant G Data Logger	Medial side of right hind leg; Lateral side of left hind leg; Lateral side of left front leg.	R	Lying	Total Lying time (Min, %) Frequency of lying bouts (number of events)	30 s or 60 s intervals	Data downloaded from Hoboware software (free) and analyzed with statistical programs (SPSS, MedCalc).	(20)
	Hobo Pendant G Data Logger	Lateral side of right hind leg	R	Lying	Lying bouts Lying time	60 s intervals	Data downloaded from Hoboware software (free) and analyzed with statistical programs (Excel).	(21)
	Afitag II	Lateral side of right hind leg	C	Lying	Lying bouts Lying time Step activity	Not specified, it is assessed that acceleration data are continuously recorded cumulatively at 15-min intervals	Automatic algorithm application (herd management software)	(21)
	SensOor	Ear (eartag)	C	Not active	Not active, Active, Highly active, Eating, Ruminating	Not specified	Automatic algorithm (unpublished) application (CowManager SensOor)	(32)
	IceQube 4hz tri-axial accelerometer	Right hind leg	C	Lying	Daily lying duration	1/s	Automatic algorithm application	(33)
	Smartbow	Ear (eartag)	C	Lying	2 posture: lying and standing or locomotion; 6 activities: milk intake, water intake, solid feed intake, ruminating, licking or sucking without milk intake, other activities.	10 Hz	Data were sent to wall-mounted receivers, which were connected to the local server on the farm where they were processed automatically by algorithm	(34)
	Hobo Pendant G Data Logger	Right hind leg	R	Lying	Lying events Standing events	1/s 1/30 s 1/min 1/2 min 1/5 min	Data downloaded from Hoboware software (free) and analyzed with statistical programs (SAS).	(35)
	Actiwatch-mini	Neck (collar)	R	Resting activity (deduced by total locomotor behaviour)	Total locomotor behaviour	32 Hz	Algorithm application	(36)
	Accelerometer designed, constructed and programmed by authors	Neck (collar)	R	Sleep	NREM sleep REM sleep Lying awake Standing	25 Hz	Wireless signal transmission, elaboration with R through a support vector machine classifier	(37)
	Hobo Pendant G Data Logger	middle occipital (placed on a halter)	R	Sleep	SLP (sleep like position) bout Daily SLP time (minutes/day) SLP bout frequency (time/day) Average bout duration (min/bout)	1/5 s	Data downloaded from Hoboware software (free) and analyzed with statistical programs (Excel, SPSS).	(38)
Machine learning with accelerometer data	Smartbow	Ear (eartag)	C	Lying	2 posture: lying and standing or locomotion; 6 activities: eating, drinking water, drinking milk, playing, ruminating, neutral/none of the above	10 Hz	Machine learning study: chaos theoretic approach	(39)
	SparkFun9, R	Neck (collar)	R	Lying	2 posture: lying and standing; 6 activities: active lying, non-active lying, locomotor play, self-grooming, non-nutritive sucking at the automatic feeder, nutritive sucking at the feeder, and ruminating	100 Hz (downsized to 50, 20, 10 4 Hz)	Machine learning study: application of classification algorithm and quantification algorithm with adjusted count method	(40)

### 3.3 Computer vision with video cameras

Two papers (41, 42) evaluated the use of computer vision technology: through this method images or video information can be analyzed to recognize and classify specific animal behaviors or postures. It must be noted that both studies evaluated the use of this technology in individually housed calves, so new perspectives in this field might be the evaluation of group-housed calves’ behaviors, in particular considering the resting pattern and social behavior of these animals.

Guo et al. (41) evaluated a new method (i.e., Integrated Background Model), built by combining background-subtraction and inter-frame difference methods to monitor the behaviors of the dairy calf. By using the new model and motion characteristics of the calf in different areas of the enclosure, the authors successfully identified the behaviors of entering the resting area, leaving the resting area, remaining stationary, turning around, feeding and drinking.

Tung et al. (42) developed a deep learning algorithm for calf posture recognition, in order to classify whether the calf is standing or lying based on images collected with cameras in two different positions, above the calf.

### 3.4 Wearable cameras

One study (43) was found through literature search for behavior identification with the use of wearable cameras, a method where the camera is attached to the animals and moves with them, circumventing identification problems and allowing a closer look for targeted behavior. This study aimed to verify if the images obtained from wearable cameras can accurately record the behavior of calves, in order to use the videos for automatic analyses using AI in future. The wearable camera was placed in a protective case and fixed to the calf’s right cheek with a commercially available calf halter. Postures such as standing and lying and behaviors such as feeding and rumination could be observed as accurately as through direct observations.

### 3.5 Real-time locating system

Ueda et al. (44) assessed the usefulness of a commercially available indoor positioning system for monitoring the resting time and moving distance in group-housed dairy calves. The method predicted lying time using the recorded displacement by IPS, a commercially available real-time locating system, including a tag (transmitter), locator (receiver), and data acquisition and processing software.

## 4 Remarkable aspects for the detection of lying, rest and sleep in dairy calves through PLF technologies

### 4.1 Tri-axial accelerometers

The application of accelerometers encompasses several critical considerations. Firstly, memory limitations are a significant factor, particularly when contrasting data loggers with wireless data acquisition systems. Data loggers possess restricted memory capacity, whereas wireless systems can transmit data in real-time and store a substantially larger volume of information.

Another relevant aspect is the impact of sampling frequencies on the accuracy of actigraphy measures. Higher sampling frequencies can provide more precise insights into specific behaviors, such as sleep, compared to more general behaviors, such as lying down. It is also essential for researchers to filter the data collected by accelerometers. This process of filtering facilitates the removal of potentially erroneous readings, thereby enhancing the overall accuracy of the measurements.

The tolerance of wearable devices is another crucial consideration. These devices must be accepted by animals without causing discomfort. For instance, devices like pedometers should not cause signs of distress, whereas ear tags were described as subject to this occurrence, requiring corrective actions.

Finally, features derived from both accelerometers and gyroscopes are indispensable for achieving high levels of accuracy in the classification of behavioral data. The integration of these features markedly improves the precision of measurements, as highlighted by Carslake et al. (40).

### 4.2 Machine learning with accelerometer data

Providing more detailed information about the level of activity performed while lying down and understanding the specific actions of the calf through data integration remains a largely unexplored area. This gap may be attributable to the challenge of identifying sufficiently sensitive sensors.

Counting behaviors that do not frequently occur based on a prediction classifier can result in overestimation. This issue is highlighted by the Classify and Count Method [as noted by Carslake et al. (40)], which fails to account for the fact that the positive predictive value decreases with prevalence. Additionally, there may be discrepancies in behavior prevalence between the training/test datasets and a new unlabeled dataset, which further complicates the accuracy of predictions.

Moreover, there is a risk of overfitting, where a model performs well on training data but fails to generalize to new data, potentially compromising the validity of the machine learning model.

### 4.3 Computer vision with video cameras

Issues encountered in recognizing calves’ behaviors were attributed to their darker images and the calf’s black and white coat. Detection challenges arose, particularly when the calf’s image overlapped with the area defined as resting zone. In their study, Guo et al. (41) utilized the average of 10 consecutive frames to derive characteristic values for behavior recognition. Consequently, instances of static behavior were occasionally misclassified as entering or leaving the resting area when the calf transitioned from a stationary state.

Moreover, the analysis of models trained on images from different cameras underscores the critical role of image quality. The findings of Tung et al. (42) emphasize that high-quality images are indispensable for enabling deep learning models to learn and accurately predict the distinctive features of calf postures.

### 4.4 Wearable cameras

Wearable cameras study had some critical outcomes related to the risk of entrapment (43), as the halter expands around the cameras. Therefore, considering their welfare, calves need to be regularly welfare-checked by an individual.

### 4.5 Real-time locating system

Ueda et al. (44) underline that while Integrated Positioning Systems (IPS) demonstrated efficacy in predicting resting time and movement distance, there is a noted need for improved accuracy in the prediction of lying time. Secondly, the effectiveness of IPS has yet to be validated in large-scale dairy farming operations with group-housed calves, necessitating further research in such settings.

## 5 Conclusions and future perspectives

Lying behavior emerged as the most frequently assessed parameter using precision livestock farming (PLF) technologies and in particular tri-axial accelerometers, highlighting its central role in research and monitoring practices. However, there is a noticeable gap in the assessment of other crucial behaviors such as resting and sleeping. These behaviors, while less frequently monitored, are essential for a comprehensive understanding of animal well-being. Evaluating the quality of rest and sleep in young animals poses significant challenges, such as the limited availability of effective monitoring tools to accurately measure and interpret these behaviors. Given the increasing application of precision livestock farming technologies for monitoring various aspects of animal health and welfare, it is essential to address the consistency of sensor-based approaches: this is particularly important when considering the sampling intervals used in accelerometer data, for example. Variability in these intervals can affect the reliability and accuracy of the collected data, underscoring the need for careful consideration and standardization in sensor methodologies. Overall, while PLF technologies offer significant advancements in monitoring animal behavior, there is a need for continued development and refinement in the methodologies employed.

The impact of rest and sleep quality on the overall welfare of dairy calves remains an area that requires more in-depth investigation, considering the importance it has for maintaining homeostasis and development. Understanding how these aspects influence calf health and development is crucial for improving welfare standards and ensuring better management practices. Moreover, several work found during the literature search and excluded for the purpose of the review have evaluated the impact of management practices, feeding, housing, sickness and pain on lying behavior: an increase of lying behavior was found in both favorable conditions (comfort) and unfavorable conditions (sickness/pain), but also to decrease in favorable conditions (like social housing - > calves are more active and play): this imply that the use of this indicator should be carefully considered and researchers should take account of multiple aspects when considering it; it is also necessary to establish threshold on healthy calves, considering the evolution in the resting-time budget during the first months of life of animals.

## Data Availability

The raw data supporting the conclusions of this article will be made available by the authors, without undue reservation.

## References

[1] European Commission D-G for H and FS (2023). Attitudes of Europeans towards animal welfare: report.

[2] Winckler C. Assessing animal welfare at the farm level: do we care sufficiently about the individual? Anim Welf. (2019) 28:77–82. doi: 10.7120/09627286.28.1.077

[3] Napolitano F Knierim U Grasso F de Rosa G. Positive indicators of cattle welfare and their applicability to on-farm protocols. Ital J Anim Sci. (2009) 8:355–65. doi: 10.4081/ijas.2009.s1.355

[4] Lawrence AB Vigors B Sandøe P. What is so positive about positive animal welfare?—a critical review of the literature. Animals. (2019) 9:783. doi: 10.3390/ani9100783, PMID: 31614498 PMC6826906

[5] Keeling LJ Winckler C Hintze S Forkman B. Towards a positive welfare protocol for cattle: a critical review of indicators and suggestion of how we might proceed. Front. Anim Sci. (2021) 2:80. doi: 10.3389/fanim.2021.753080, PMID: 39651492

[6] Mattiello S Battini M De Rosa G Napolitano F Dwyer C. How can we assess positive welfare in ruminants? Animals. (2019) 9:758. doi: 10.3390/ani9100758, PMID: 31581658 PMC6826499

[7] Papageorgiou M Simitzis PE. Positive welfare indicators in dairy animals. Dairy. (2022) 3:814–41. doi: 10.3390/dairy3040056

[8] Sutherland MA Stewart M Schütz KE. Effects of two substrate types on the behaviour, cleanliness and thermoregulation of dairy calves. Appl Anim Behav Sci. (2013) 147:19–27. doi: 10.1016/j.applanim.2013.04.018

[9] Rechtschaffen A. Current perspectives on the function of sleep. Perspect Biol Med. (1998) 41:359–90. doi: 10.1353/pbm.1998.0051, PMID: 9604368

[10] Siegel JM. Clues to the functions of mammalian sleep. Nature. (2005) 437:1264–71. doi: 10.1038/nature04285, PMID: 16251951 PMC8760626

[11] Steiger A. Sleep and the hypothalamo-pituitary-adrenocortical system. Sleep Med Rev. (2002) 6:125–38. doi: 10.1053/smrv.2001.0159, PMID: 12531148

[12] Morrissey MJ Duntley SP Anch AM Nonneman R. Active sleep and its role in the prevention of apoptosis in the developing brain. Med Hypotheses. (2004) 62:876–9. doi: 10.1016/j.mehy.2004.01.014, PMID: 15142640

[13] Panivivat R Kegley EB Pennington JA Kellogg DW Krumpelman SL. Growth performance and health of dairy calves bedded with different types of materials. J Dairy Sci. (2004) 87:3736–45. doi: 10.3168/jds.S0022-0302(04)73512-2, PMID: 15483157 PMC7190087

[14] Hänninen L De Passillé AM Rushen J. The effect of flooring type and social grouping on the rest and growth of dairy calves. Appl Anim Behav Sci. (2005) 91:193–204. doi: 10.1016/j.applanim.2004.10.003

[15] Camiloti TV Fregonesi JA von Keyserlingk MAG Weary DM. Short communication: effects of bedding quality on the lying behavior of dairy calves. J Dairy Sci. (2012) 95:3380–3. doi: 10.3168/jds.2011-5187, PMID: 22612971

[16] Neja W. Behaviour of calves in the first weeks of life. J Cent Eur Agric. (2013) 14:33–41. doi: 10.5513/JCEA01/14.1.1151

[17] Sutherland MA Worth GM Cameron C Ross CM Rapp D. Health, physiology, and behavior of dairy calves reared on 4 different substrates. J Dairy Sci. (2017) 100:2148–56. doi: 10.3168/jds.2016-12074, PMID: 28109608

[18] O’Driscoll K Von Keyserlingk MAG Weary DM. Effects of mixing on drinking and competitive behavior of dairy calves. J Dairy Sci. (2006) 89:229–33. doi: 10.3168/jds.S0022-0302(06)72087-2, PMID: 16357286

[19] Hill TM Bateman HG Aldrich JM Quigley JD Schlotterbeck RL. Short communication: intensive measurements of standing time of dairy calves housed in individual pens within a naturally ventilated, unheated nursery over different periods of the year. J Dairy Sci. (2013) 96:1811–4. doi: 10.3168/jds.2012-6206, PMID: 23332839

[20] Bonk S Burfeind O Suthar VS Heuwieser W. Technical note: evaluation of data loggers for measuring lying behavior in dairy calves. J Dairy Sci. (2013) 96:3265–71. doi: 10.3168/jds.2012-6003, PMID: 23498014

[21] Swartz TH McGilliard ML Petersson-Wolfe CS. Technical note: the use of an accelerometer for measuring step activity and lying behaviors in dairy calves. J Dairy Sci. (2016) 99:9109–13. doi: 10.3168/jds.2016-11297, PMID: 27614829

[22] Hart BL. Biological basis of the behavior of sick animals. Neurosci Biobehav Rev. (1988) 12:123–37. doi: 10.1016/S0149-7634(88)80004-6, PMID: 3050629

[23] Borderas TF De Passillé AM Rushen J. Behavior of dairy calves after a low dose of bacterial endotoxin. J Anim Sci. (2008) 86:2920–7. doi: 10.2527/jas.2008-0926, PMID: 18641175

[24] Cantor MC Costa JHC. Daily behavioral measures recorded by precision technology devices may indicate bovine respiratory disease status in preweaned dairy calves. J Dairy Sci. (2022) 105:6070–82. doi: 10.3168/jds.2021-20798, PMID: 35282905

[25] Veissier I Le Neindre P Trillat G. The use of circadian behaviour to measure adaptation of calves to changes in their environment. Appl Anim Behav Sci. (1989) 22:1–12. doi: 10.1016/0168-1591(89)90075-0

[26] von Keyserlingk MAG Cunha GE Fregonesi JA Weary DM. Introducing heifers to freestall housing. J Dairy Sci. (2011) 94:1900–7. doi: 10.3168/jds.2010-3994, PMID: 21426979

[27] Ruckebusch Y. The relevance of drowsiness in the circadian cycle of farm animals. Anim Behav. (1972) 20:637–43. doi: 10.1016/S0003-3472(72)80136-2, PMID: 4661312

[28] Hänninen L Mäkelä JP Rushen J de Passillé AM Saloniemi H. Assessing sleep state in calves through electrophysiological and behavioural recordings: a preliminary study. Appl Anim Behav Sci. (2008) 111:235–50. doi: 10.1016/j.applanim.2007.06.009

[29] Tucker CB Jensen MB de Passillé AM Hänninen L Rushen J. Invited review: lying time and the welfare of dairy cows. J Dairy Sci. (2021) 104:20–46. doi: 10.3168/jds.2019-18074, PMID: 33162094

[30] Stygar AH Gómez Y Berteselli GV Dalla Costa E Canali E Niemi JK . A systematic review on commercially available and validated sensor Technologies for Welfare Assessment of dairy cattle. Front Vet Sci. (2021) 8:634338. doi: 10.3389/fvets.2021.634338, PMID: 33869317 PMC8044875

[31] Trénel P Jensen MB Decker EL Skjøth F. Technical note: quantifying and characterizing behavior in dairy calves using the IceTag automatic recording device. J Dairy Sci. (2009) 92:3397–401. doi: 10.3168/jds.2009-2040, PMID: 19528617

[32] Hill TM Suarez-Mena FX Hu W Dennis TS Schlotterbeck RL Timms LL . TECHNICAL NOTE: evaluation of an ear-attached movement sensor to record rumination, eating, and activity behaviors in 1-month-old calves. Prof Anim Sci. (2017) 33:743–7. doi: 10.15232/pas.2017-01623

[33] Finney G Gordon A Scoley G Morrison SJ. Validating the IceRobotics IceQube tri-axial accelerometer for measuring daily lying duration in dairy calves. Livest Sci. (2018) 214:83–7. doi: 10.1016/j.livsci.2018.05.014

[34] Roland L Schweinzer V Kanz P Sattlecker G Kickinger F Lidauer L . Technical note: evaluation of a triaxial accelerometer for monitoring selected behaviors in dairy calves. J Dairy Sci. (2018) 101:10421–7. doi: 10.3168/jds.2018-14720, PMID: 30146297

[35] Rivera-Barreto M Colón-Rodríguez I Soriano-Varela G Golderos-Trujillo C Maeso-Ramírez N Torres-Sifre M . Evaluation of a commercial accelerometer for remote monitoring of lying and standing events in dairy calves in Puerto Rico. J Agric Univ Puerto Rico. (2020) 104:31–42. doi: 10.46429/jaupr.v104i1.18286

[36] Giannetto C Cerutti RD Scaglione MC Arfuso F Pennisi M Giudice E . Real-time measurement of the daily Total locomotor behavior in calves reared in an intensive management system for the possible application in precision livestock farming. Vet Sci. (2023) 10:64. doi: 10.3390/vetsci10010064, PMID: 36669065 PMC9866244

[37] Hokkanen AH Hänninen L Tiusanen J Pastell M. Predicting sleep and lying time of calves with a support vector machine classifier using accelerometer data. Appl Anim Behav Sci. (2011) 134:10–5. doi: 10.1016/j.applanim.2011.06.016

[38] Fukasawa M. The development of sleep-like posture expression with age in female Holstein calves. Anim Sci J. (2023) 94:e13816. doi: 10.1111/asj.13816, PMID: 36802332

[39] Sturm V Efrosinin D Efrosinina N Roland L Iwersen M Drillich M . A Chaos theoretic approach to animal activity recognition. J Math Sci. (2019) 237:730–43. doi: 10.1007/s10958-019-04199-9

[40] Carslake C Vázquez-Diosdado JA Kaler J. Machine learning algorithms to classify and quantify multiple behaviours in dairy calves using a sensor–moving beyond classification in precision livestock. Sensors. (2021) 21:1–14. doi: 10.3390/s21010088, PMID: 33375636 PMC7795166

[41] Guo Y He D Chai L. A machine vision-based method for monitoring scene-interactive behaviors of dairy calf. Animals. (2020) 10:190. doi: 10.3390/ani10020190, PMID: 31978962 PMC7071125

[42] Tung TC Khairuddin U Shapiai MI Nor NM Hiew MWH Suhaimie NAM. Calf posture recognition using convolutional neural network. Comput Mater Con. (2023) 74:1493–508. doi: 10.32604/cmc.2023.029277, PMID: 39055887

[43] Saitoh T Kato Y. Evaluation of wearable cameras for monitoring and analyzing calf behavior: a preliminary study. Animals. (2021) 11:2622. doi: 10.3390/ani11092622, PMID: 34573586 PMC8470911

[44] Ueda K Ishii T Nakai Y Odaka K. Use of a commercial indoor positioning system for monitoring resting time and moving distance in group-housed dairy calves. Anim Sci J. (2023) 94:e13830. doi: 10.1111/asj.13830, PMID: 36992544

